# Editorial: Economic Effects of COVID-19 Related Uncertainty Shocks

**DOI:** 10.3389/fpubh.2021.760619

**Published:** 2021-09-28

**Authors:** Giray Gozgor, Chi Keung Marco Lau

**Affiliations:** ^1^Faculty of Political Sciences, Istanbul Medeniyet University, Istanbul, Turkey; ^2^Business School, Teesside University, Middlesbrough, United Kingdom

**Keywords:** outcomes of the COVID-19 pandemic, economic effects of the COVID-19, effects of the COVID-19 pandemic on financial market, effects of pandemics on businesses, COVID-19 related uncertainty shocks

In recent years, the world economy has also witnessed the rise in economic and policy uncertainty due to Brexit, the deceleration of growth in the Chinese economy, the Trump administration's trade policy. There are now more massive uncertainty shocks of the COVID-19 in every aspect of the economic system. Both developed and developing economies are experiencing risks and uncertainties due to the significant effects of the COVID-19. This Research Topic aims to understand the consequences of uncertainty shocks related to the COVID-19. The main objective of this Research Topic is to try to provide different aspects and consequences of the COVID-19 pandemic. The Research Topic contains 26 papers.

This Research Topic includes several papers on the determinants and the outcomes of the COVID-19 pandemic. Gorain et al. discuss the fighting strategies against the COVID-19 pandemic at the global level. Meng observe the different country clusters for the measures of the COVID-19 outcomes in the G20 economies. Jiang et al. find that the health levels in the United States and the United Kingdom are not significantly affected by the COVID-19-related shocks. Zhai et al. observe that conflicts are the main driver of the mortality risk of the COVID-19 in 120 countries. Wang et al. show that the COVID-19 pandemic causes geopolitical risks in 18 emerging economies. Fang et al. use the trend analysis data from the Baidu search engine to predict the pattern of the COVID-19 pandemic in China.

The Research Topic also covers several papers on the economic effects of the COVID-19 pandemic. Wu finds that pandemics-related uncertainty, measured by the Pandemic Uncertainty Index, negatively affects household consumption in 138 countries. Li and Liang observe that uncertainties related to the COVID-19, measured by the World Pandemic Uncertainty Indices, are positively related to fiscal support in 129 countries. Li Y. et al. discuss the efficiency of monetary policy implications in Brazil, China, India, Japan, and the United Kingdom during the pandemics. Chen, Lau et al. show that the pandemics-related uncertainty is positively related to the income inequality in 34 OECD economies, but the impact is negative in 107 non-OECD countries. Regarding China, where the COVID-19 pandemic first hit the economy, Shen et al. observe the significant divergence of the trend of disposable income across the Chinese cities during the COVID-19 pandemic. Li J. et al. also predict that the monthly income of migrant workers in China will be reduced due to the COVID-19 pandemic.

This Research Topic also includes several papers on the effects of the COVID-19 pandemic on financial markets. Liu and Guo show the positive impact of inclusive finance on public health in the Chinese regions during the pandemics. Pan et al. examine the possible determinants of tourism stock returns in China during the COVID-19 era. Ye et al. compare different models to estimate the equity performances of the health insurance companies in China during the COVID-19 pandemic. Meng et al. observe that the confirmed cases cause public searches for the COVID-19 tests, which also causes fluctuations in the infectious disease equity market volatility tracker in the United States. Wang et al. also find that the United States Dollar (USD) and the volatility indices (VIX) decrease the S&P 500 equity returns during the COVID-19 crisis. However, the newspaper-based infectious disease equity market volatility tracker is positively associated with stock market returns in the United States. Chen, Gozgor et al. also show that the economic policy uncertainty in China has a positive impact on the returns of Bitcoin during the COVID-19 era, i.e., from December 31, 2019 to May 20, 2020. Algamdi et al. find that the COVID-19 related deaths have negatively affect crude oil prices, and this evidence has provided economic implications on Saudi Arabia and the United States.

Finally, the Research Topic covers the effects of the COVID-19 pandemic and other pandemics on businesses. Su and Zhou use the case of Chongqing (China) to discuss the ecological livability of tourist towns with the outbreak of the COVID-19 pandemic. Limei and Wei take online travel booked by consumers as an example and consider Guangzhou Baiyun International Airport (China) data to analyze the influence of reviewers' creditworthiness on consumer purchase intention. Determinants of purchase intention provide several implications to be used during the COVID-19 pandemic. Song et al. evaluate the impact of the COVID-19 pandemic on China's manufacturing sector in the global value chain. Cao discusses the determinants of mobile payment adoption, which is expected to rise during the COVID-19 era for small and medium enterprises in China. Ai and Peng observe that dynamic capabilities, intra-industry networks, and social capital are the main determinants of the innovation dynamics of Chinese firms, and this evidence provides several implications for the COVID-19 crisis. In terms of global datasets, Zhu et al. show that pandemics-related uncertainty, measured by the World Pandemic Uncertainty Index, is positively related to socially responsible investments. Finally, Shang et al. provide a business history review on the effects of pandemics on economic performance.

Overall, this Research Topic covers 26 papers on the determinants and the outcomes of the COVID-19 pandemic, economic aspects of the COVID-19 pandemic and other pandemics, effects of the COVID-19 pandemic on financial markets, and the effects of COVID-19 pandemic and other pandemics on businesses.

## Author Contributions

All authors listed have made a substantial, direct and intellectual contribution to the work, and approved it for publication.

## Conflict of Interest

The authors declare that the research was conducted in the absence of any commercial or financial relationships that could be construed as a potential conflict of interest.

## Publisher's Note

All claims expressed in this article are solely those of the authors and do not necessarily represent those of their affiliated organizations, or those of the publisher, the editors and the reviewers. Any product that may be evaluated in this article, or claim that may be made by its manufacturer, is not guaranteed or endorsed by the publisher.

